# Health Outcomes with Curative and Palliative Therapies in Real World: Role of the Quality of Life Summary Score in Thoracic Oncology Patients

**DOI:** 10.3390/cancers15153821

**Published:** 2023-07-27

**Authors:** Kurt G. Tournoy, Valerie Adam, Inge Muylle, Helene De Rijck, Ellen Everaert, Ehsan Eqlimi, Jan P. van Meerbeeck, Piet Vercauter

**Affiliations:** 1 Department of Respiratory Medicine, Onze-Lieve Vrouw Ziekenhuis, 9300 Aalst, Belgium; valerie.adam@olvz-aalst.be (V.A.); inge.muylle@olvz-aalst.be (I.M.); leen.derijck@olvz-aalst.be (H.D.R.); ellen.everaert@olvz-aalst.be (E.E.); piet.vercauter@olvz-aalst.be (P.V.); 2 Faculty of Medicine and Life Sciences, Ghent University, 9000 Ghent, Belgium; 3 Clinical Trial Center and Center of Biostatistics, Onze-Lieve Vrouw Ziekenhuis, 9300 Aalst, Belgium; ehsan.eqlimi@olvz-aalst.be; 4 Department of Respiratory Medicine, Antwerp University Hospital, 2650 Edegem, Belgium; jan.vanmeerbeeck@uza.be

**Keywords:** lung cancer, value-based health care, quality of life, QLQ-C30 questionnaire, quality of life summary score, global health score, WHO performance score, thoracic neoplasms, curative therapy, palliative therapy

## Abstract

**Simple Summary:**

For patients receiving therapy with curative or palliative intent for a thoracic malignancy, prediction of how the quality of life evolves once therapy starts remains challenging. The role of health assessments by the patient instead of the physician herein is ill-defined. We show that patients with thoracic malignancies treated with curative intent experience a worsening of their health in the first year, whereas those receiving palliative anti-cancer therapy do not. Health scores reported by the patient are multidimensional, but can now be condensed into a summary score. Health, one year after the start of therapy, can partly be predicted by the baseline health summary score as determined by the patient, the comorbidity burden, and the therapeutic strategy. Our data, therefore, support the assessment of the health by the patients rather than by the physician as it provides useful predictive information for the health one year after the start of the cancer therapy.

**Abstract:**

Background: For patients receiving therapy with curative or palliative intent for a thoracic malignancy, prediction of quality of life (QOL), once therapy starts, remains challenging. The role of health assessments by the patient instead of the doctor herein remains ill-defined. Aims: To assess the evolution of QOL in patients with thoracic malignancies treated with curative and palliative intent, respectively. To identify factors that determine QOL one year after the start of cancer therapy. To identify factors that affect survival. Methods: We prospectively included consecutive patients with a thoracic malignancy who were starting anti-cancer therapy and measured QOL with QLQ-C30 before the start of therapy, and thereafter at regular intervals for up to 12 months. A multivariate regression analysis of the global health score (GHS) and QOL summary scores (QSS) one year after the start of therapy was conducted. A proportional hazards Cox regression was conducted to investigate the effects of case-mix variables on survival. Results: Of 587 new patients, 375 started different forms of therapy. Most had non-small cell lung cancer (*n* = 298), 35 had small cell lung cancer, and 42 had other thoracic malignancies or were diagnosed on imaging alone. There were 203 who went for a curative intent and 172 for a palliative intent strategy. The WHO score of 0–1 was more prevalent in the former group (*p* = 0.02), and comorbidities were equally distributed. At baseline, all QOL indices were better in the curative group (*p* < 0.05). The curative group was characterized by a significant worsening of GHS and QSS (*p* < 0.05). The palliative group was characterized by an improvement in GHS and emotional health (*p* < 0.05), while other dimensions of functioning remained stable. GHS at 12 months was estimated in a multivariate linear regression model (R^2^ = 0.23—*p* < 0.001) based on baseline GHS, QSS, and comorbidity burden. QSS at 12 months was estimated (R^2^ = 0.31—*p* < 0.001) by baseline QSS and therapeutic intent strategy (curative vs. palliative). The prognostic factors for overall survival were the type of therapy (curative vs. palliative intent, *p* < 0.001) and occurrence of early toxicity-related hospitalization (grade ≥ 3, *p* = 0.001). Conclusion: Patients with thoracic malignancies treated with curative intent experience a worsening of their QOL in the first year, whereas those receiving palliative anti-cancer therapy do not. QOL one year after the start of therapy depends on the baseline health scores as determined by the patient, comorbidity burden, and therapeutic strategy. Survival depends on therapeutic strategy and early hospitalization due to toxicity.

## 1. Introduction

The decision to consider anti-cancer therapy for patients with thoracic malignancies is based on the integration of many objective and subjective factors [1,2]. Typically, tumor type and its molecular characteristics, stage and related therapeutic strategy (palliative vs. curative), comorbidity burden, and the World Health Organization (WHO) score are identified as prognostic factors for thoracic malignancies and guide treatment allocation [3,4,5]. The treating physician estimates these factors. Other factors such as social context and expectations of the patient and his/her close ones and the health status as assessed by the patient are—though more difficult to handle—also of potential importance. 

When a patient faces the diagnosis and treatment of a thoracic malignancy, uncertainty prevails about the impact on his/her future quality of life (QOL). The most common tool to assess the patient’s condition or health status to tolerate therapies is the performance score, according to the WHO or Karnofsky score [6]. This is easy to use and has repeatedly shown prognostic value [7]. However, as a categorical variable, the WHO score is not very balanced and ignores the input of the patient. Only a moderate level of agreement between the healthcare provider and lung cancer patient recorded health status exists, with the former systematically granting better scores [8,9,10]. This makes the performance score potentially less accurate in describing health and, more importantly, in predicting its evolution upon cancer therapy. 

The QLQ-C30 is a patient-reported assessment of health. It can be deployed at baseline, but also to monitor the evolution after starting therapy. It encompasses a global health score (GHS), five functional and nine symptom scores, the latter two summarized in a QOL-Summary Score (QSS) [11]. With the QSS ranging from 0 to 100, a higher score denoting a better QOL, it holds the promise as a valuable and better-balanced tool compared to the performance score. The question thus arises whether it is of added value for the care of patients with thoracic cancers, especially to monitor the health status, but also as a covariate to predict future health status. 

Therefore, we prospectively evaluated the health status during either curative or palliative therapy in a large group of patients presenting with thoracic malignancy. We document the QOL encompassing GHS and QSS, but also the different dimensions of the latter. We analyzed if GHS and QSS codetermine survival in addition to other patient variables, including comorbidity burden, performance status, therapeutic strategy, unforeseen therapy-related hospitalization, and age. Finally, we deploy a multivariable regression model to unveil which factors determine the QOL 12 months after therapy start and analyze if baseline GHS and QSS harbor valuable information for treatment allocation. 

## 2. Materials and Methods

### 2.1. Population Selection

Patients in whom a primary thoracic malignancy (lung cancer, mesothelioma, neuro-endocrine tumors) was diagnosed and who were amenable to anti-cancer therapy in the department of pulmonary medicine and thoracic oncology of Onze-Lieve-Vrouw Hospital Aalst-Belgium were eligible for this prospective study. Patients had to give written informed consent for the current study before the start of the therapy. This prospective single-center academic study was registered and approved by the local ethics committee of OLV-Aalst Hospital in Belgium (registry nr 2017/101-Belgian Registration nr B126201734031).

### 2.2. Acquisition of Data

Baseline clinical data (sex, age, body mass index (BMI), smoking status, performance score, comorbidities, cancer subtype, TNM-8 stage, and therapy characteristics) were collected from the electronic medical file by the oncology nurse. Comorbidities were assessed using the modified Self-reported Comorbidity Questionnaire [12,13]. The patient indicated the presence or absence of the 12 items. If necessary, the data nurse completed these based on the available medical history. We defined two anti-cancer therapy intents: curative and palliative. Patients going for either (i) primary thoracic surgery (*w*/*wo* adjuvant therapies), (ii) primary radical thoracic radiation therapy, or (iii) combined modality curative therapies (based on any combination of chemotherapy with either radiotherapy and/or surgery *w*/*wo* adjuvant therapies) were included in the group with curative intent. The group with palliative intent encompassed patients scheduled for either palliative chemotherapy, targeted therapy, immunotherapy, palliative radiation therapy, or any combination of these. Severe complications were defined as unforeseen hospitalizations related to the therapy. Common Terminology Criteria for Adverse Events (CTCAE v5.0) toxicity characteristics were used to define Grade 3–4 toxicities of systemic and radiation therapy. The presence or absence of hospitalization due to therapy toxicity within 3 months after therapy start defined this variable. 

Tumor responses were assessed by chest CT every 2–3 months in the first year or whenever there was a clinical need. Survival characteristics were estimated with the Kaplan–Meier and log-rank (Mantel–Cox) methods, the latter for multivariable analysis. The date of pathological diagnosis was the start date (or the date of the multidisciplinary board where a diagnosis was assigned in case pathology was not available) to estimate follow-up until progression or death (in months). To ensure data maturity, we followed all patients for at least 18 months after the start of therapy. A data lock was set on 31 January 2023. 

### 2.3. Patient-Reported Outcomes (PROs)

Questionnaires to gauge the quality of health were available for the patients in the days before a consultation in the thoracic oncology clinic was foreseen. The patient was asked to complete it on his/her cell phone or home computer. Otherwise, a paramedical team member offered the questionnaire on a tablet just prior to the patient’s visit to the thoracic oncologist. The patient completed the questionnaire either alone or assisted by family members or a paramedic whenever needed. When patients had progressive disease or were too weak to continue PROs, the recording was discontinued. We selected the generic QOL Questionnaire Core 30 (QLQ-C30, version 3.0, available at https://qol.eortc.org/questionnaire/eortc-qlq-c30/, accessed on 14 November 2017) as a validated tool to measure subjective health outcomes. It encompasses (i) global QOL, (ii) functional scores (FS), and (iii) symptom scores (SS) [13]:

- Global QOL is estimated by global health score (GHS) and is based on two questions (score range 1–7). A higher GHS (range 0–100) means a better global QOL;
- The FS is composed of five domains, each with a 4-point score range: (a) physical, (b) role, (c) emotional, (d) cognitive, and (e) social functioning. These FSs are estimated by two questions per status, except physical functioning, which is assessed by five questions. A higher calculated FS score means a better status (range 0–100);
- The SS measures cancer-specific symptoms by 1 or 2 questions on a 4-point score scale. The higher the calculated symptom score (range 0–100), the more complaints the patient has.

The FS and SS (except financial burden) were combined into a quality of life summary score (QSS) by averaging, but after reversing the symptom scores so a higher score means a lower symptom burden [11]. Compliance was calculated as a ratio of the number of patients that completed the questionnaires over the number of survivors at the time point it needed to be completed. QOL questionnaires were completed in the first year of this project and offered at baseline, three, six, and twelve months only to those patients going for curative therapy. Thereafter, the QOL questionnaires were offered to all and with two additional assessments at 6 weeks and 9 months. As a consequence, a difference exists between the population size (*n* = 375) and the number of health measurements available at baseline (*n* = 303). We calculated the changes versus baseline measurement to document the evolution of the QOL scores. Changes of 10 points or greater have been accepted as clinically meaningful; however, differences as low as 4 points have been reported to be relevant in lung cancer trials [14,15]. We based our conclusions on those differences that reached significance after statistical testing. 

### 2.4. Statistical Analysis 

Data were analyzed with SPSS 28.0 (IBM Corp. Released 2021. IBM SPSS Statistics for Windows, Version 28.0. IBM Corp: Armonk, NY, USA). Continuous variables are presented as means ± standard error of means (SEM) unless otherwise indicated. The distribution of categorical variables is presented as counts and as a percentage of the selected population. Baseline health scores were compared with unpaired *t*-tests between the curative and palliative groups. Proportions were compared using the χ^2^-test. The evolution of health scores over time was estimated by calculation of the difference at each time point versus baseline and compared with paired *t*-tests (with the exclusion of cases analysis by analysis when missing values occurred) with Bonferroni adjustment. For the multivariable regression models of GHS and QSS at 12 months, the assumptions of normality, linearity, homoscedasticity, and absence of collinearity were tested. For the detection of the prognostic variables, a Cox regression analysis for survival was run with both continuous and (transformed) bivariate parameters. We did not perform data imputation to handle missing data. A two-sided significance level at *p* < 0.05 was taken for all tests. 

## 3. Results

### 3.1. Patient Characteristics

**Figure 1:**
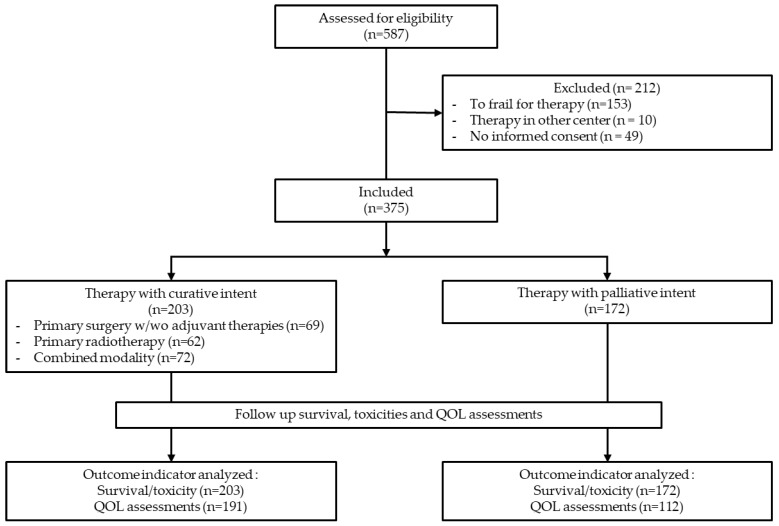
Overview of the study population. With or without (*w*/*wo*).

From January 2018 until April 2021, 587 new patients were diagnosed with a thoracic malignancy (Figure 1, consort diagram). Of these, 212 (36%) did not enter the health quality project: 153 (26%) were too frail to start any oncology therapy and received only supportive care, 59 (10%) were not included because of various reasons (therapy in another center, absence of informed consent or therapy start before a baseline measurement was conducted). So, 375 newly diagnosed patients starting either curative (*n* = 203) or palliative cancer therapy (*n* = 172) were included. 

The majority had non-small cell lung cancer (NSCLC, *n* = 298, 80%) or small cell lung cancer (SCLC, *n* = 35, 9%), although 42 had other thoracic malignancies (e.g., mesothelioma or carcinoids; *n* = 14, 4%) or no formal pathological proof of malignancy (*n* = 28, 7%). Of the 107 patients with stage IV non-squamous lung carcinoma, molecular analysis was available for 103 (96%) and showed EGFR mutations in 18 (17%), ALK-EML4 fusion in 1 (1%), and BRAF in 3 (3%), while KRAS was found in 36 (35%). 

Table 1 summarizes the characteristics of both therapy groups. The group treated with curative intent was characterized by a higher BMI (*p* = 0.002) and a better performance score with more patients in the WHO 0–1 (*p* = 0.02) as compared to those treated with palliative intent. There were also more patients in whom no pathological confirmation of lung cancer was obtained, despite going for curation (*p* < 0.001). This is explained by a subgroup receiving curative radiation therapy in the absence of pathological proof of malignancy. The comorbidity burden was equally distributed between both groups. Patients going for curation (*n* = 203) had stage I–III disease and were treated by surgery *w/wo* adjuvant therapies (*n* = 69), primary radiotherapy (*n* = 62), or combined modality therapies (*n* = 72). Those starting palliative therapy (*n* = 172) were mainly stage IV patients, although some (*n* = 7) had stage III disease. 

### 3.2. Health Status as Reported by the Patient per Group

**Figure 2:**
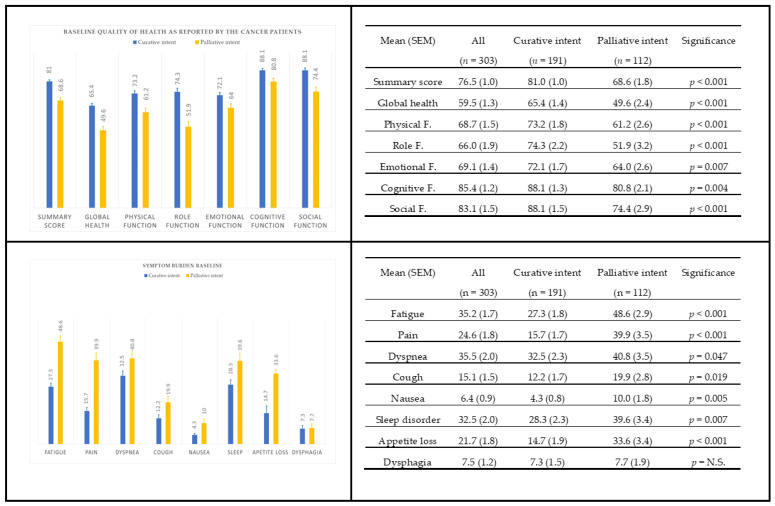
Baseline health status of patients. Data are presented as mean scores (±SEM). The significance level refers to the comparison of the health scores between curative and palliative groups. In the first year of the project, health measurements were offered only to those going for curative therapy, explaining the difference in health measurements (*n* = 303) as compared to the total number of patients included (*n* = 375). Not Significant (N.S.).

There were 303 patients in the QOL assessment database; 191 received curative therapy, and 112 received palliative therapy (Figure 2). At baseline, compliance for completing the QOL forms was 100%, while at 6 and 12 months, this was 90% and 93%, respectively. Patients had a better quality of life before the start of therapy with curative intent as compared to palliative therapy based on GHS and QSS (*p* < 0.05), but also on all individual FS domains and SS, except dysphagia. A point biserial correlation was run to measure the strength and direction of the association between the WHO category (0–1 and 2–3) vs. both baseline GHS and QSS. The Pearson correlation was −0.22 (95%CI −0.33 to −0.11) and −0.30 (95%CI −0.40 to −0.20), respectively (*p* < 0.001 for both); a higher WHO score indicates a lower GHS or QSS at baseline.

**Figure 3:**
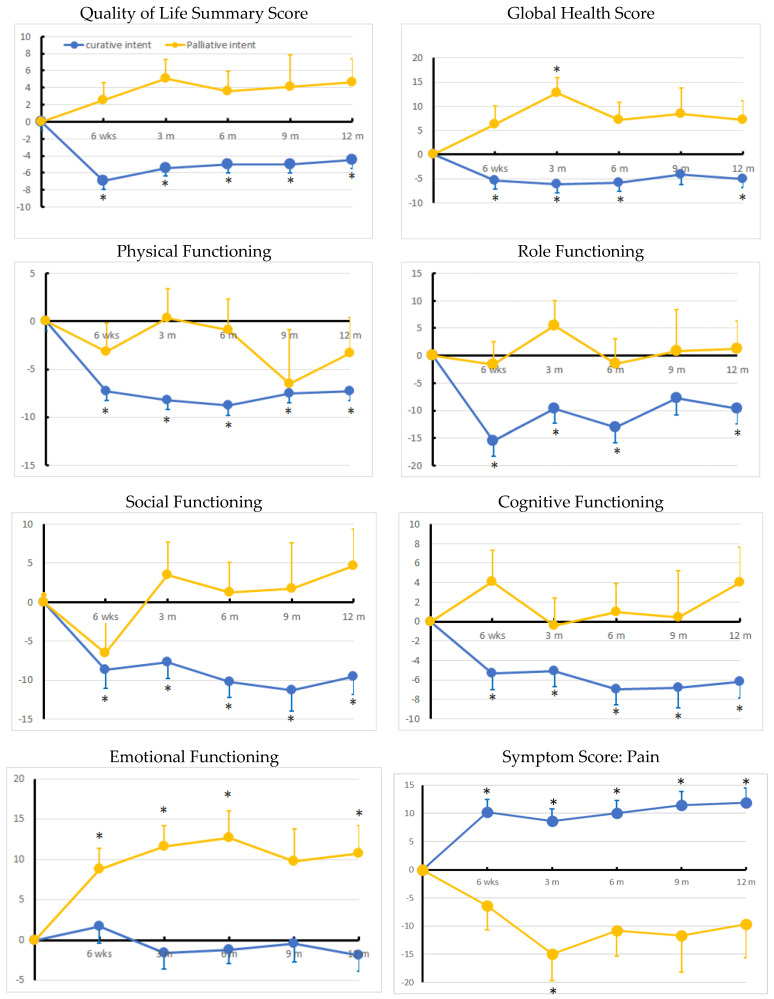
Evolution Quality of Life scores (QSS, GHS, FS, and pain). Curative is blue, and palliative is yellow. Values are the difference as compared to the baseline value. Significant changes (*p* < 0.05) are indicated with an asterisk. For further details, see Supplementary Table S1.

The evolution of the QOL over a one-year period after a cancer diagnosis is depicted per group in Figure 3 and is further detailed in Table S1 (upper panel). The curative group was characterized by an immediate and persistent significant deterioration in GHS and QSS (*p* < 0.05 vs. baseline at all time points, except for GHS at 9 months after Bonferroni’s adjustment). All FS except emotional functioning worsened from 6 weeks onwards and did not recover to the baseline value (*p* < 0.05 for all time points, except for role functioning at 9 months). For symptom burden (see Table S1, lower panel), a persistent increase in fatigue and pain was seen. Loss of appetite was found only at 6 weeks, but not thereafter. In this group, there was no indication of improvement for any of the dimensions measured at any time point. 

The palliative group was characterized by a significant and persistent improvement in the emotional functioning domain (*p* < 0.05 vs. baseline at all time points, except at 9 months), while GHS showed significant improvements only at 3 months. Sleep disorders did improve significantly, as did pain, although not persistently. 

### 3.3. Univariable and Multivariable Cox Regression Model to Predict Survival

**Figure 4:**
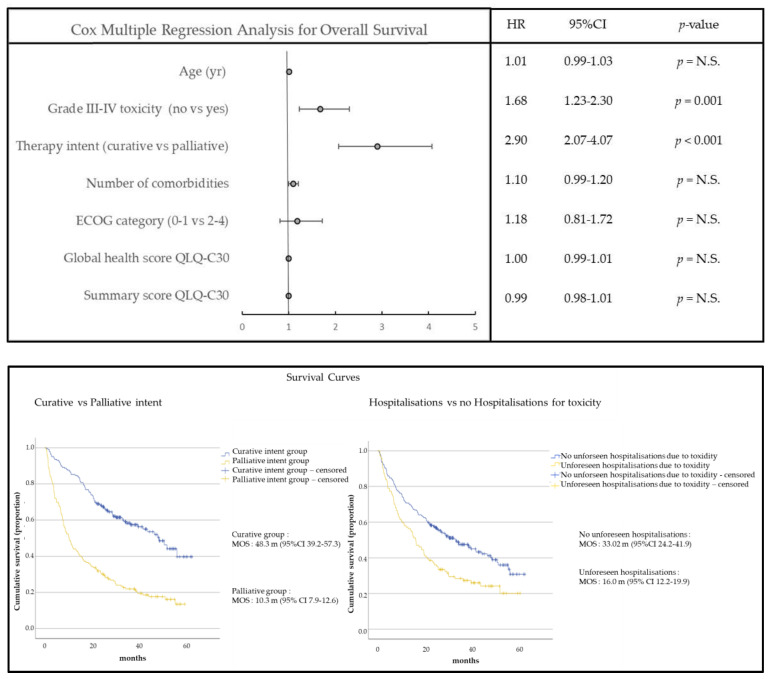
Cox Regression Analysis for survival. (**Upper panel**): Forest plot summarizing the Cox Regression analysis deployed for the determination of those factors that contribute to overall survival. A Hazard ratio above 1.0 means that (a higher) exposure to the continuous or bivariate variable increases the risk of death. (**Lower panel**): Survival curves for therapy strategy (curative vs. palliative—left) and for the presence or absence of unforeseen hospitalizations due to toxicity (right) analyzed by the Kaplan–Meier method. MOS: median overall survival (months). Not Significant (N.S.).

Figure 4 shows the forest plot that identifies factors determining the survival of the cohort. The median overall survival of the group treated with curative intent was 48.3 months (95% CI 39.2–57.3). In the group with palliative therapies, this was 10.3 months (95% CI 7.9–12.6) (Log-Rank *p* < 0.001). In univariate analysis, all considered variables reached statistical significance. In a multivariable Cox regression model, only the therapeutic strategy (HR 2.90, 95% CI 2.07–4.07; *p* < 0.001) and the occurrence of severe non-hematologic toxicity (HR 1.68, 95% CI 1.23–2.30; *p* = 0.001) were prognostic, while baseline QSS, GHS, WHO performance, comorbidity burden, and age were not. A sensitivity analysis selected only on patients with non-small cell lung cancer yielded similar results. 

### 3.4. Multilinear Regression Model for GHS and QSS One Year after Diagnosis

**Figure 5:**
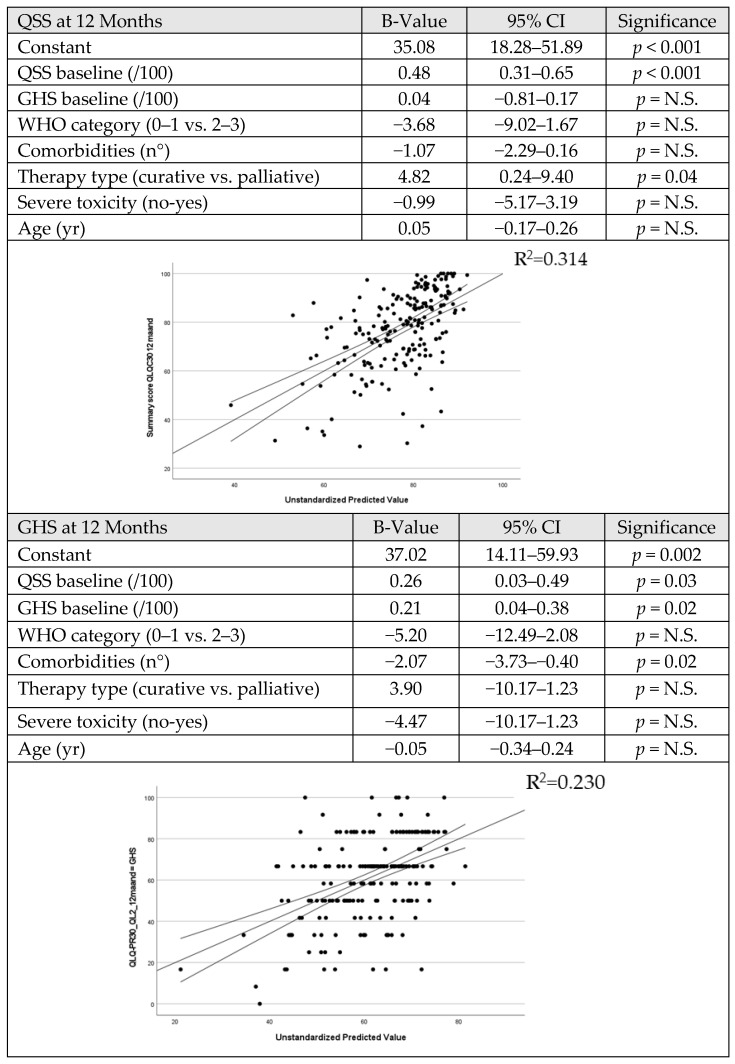
Multivariable regression to predict QOL 12 months after therapy start. (**Upper panel**) QSS at 12 months; (**lower panel**) GHS at 12 months. Not Significant (N.S.).

We found that the baseline QSS (*p* < 0.001) and therapeutic strategy (*p* = 0.04) significantly contributed to the estimation of the QSS at 12 months. The WHO score, baseline GHS, comorbidity burden, toxicity, and age did not (Figure 5 upper panel). For every 1-point higher baseline QSS, the QSS at 12 months increased by 0.5 points. The strength of the model overall was moderate, with a regression coefficient of R = 0.56 (R^2^ = 0.31, *p* < 0.001). 

For the estimation of GHS at 12 months (see Figure 5—lower panel), we found that baseline QSS (*p* = 0.03), baseline GHS (*p* = 0.02), and the comorbidity burden (*p* = 0.02) significantly contributed. The WHO score, therapeutic strategy, toxicity, or age did not. For every 1-point higher baseline QSS and GHS, the GHS at 12 months increased by 0.3 and 0.2 points. Per extra comorbidity, the patient loses 2.1 GHS points after 12 months. The strength of this model was weak, with a regression coefficient of R = 0.48 (R^2^ = 0.23, *p* < 0.001).

## 4. Discussion

The most important conclusion from this study is that patients with a thoracic malignancy who are treated with curative intent experience a solid and long-lasting decline in QOL (represented by both GHS and QSS). This contrasts with observations for palliative therapy in which patients indicate a stabilization and some improvements. The QOL after one year strongly depends on the health status before starting therapy as assessed by the patient rather than by the oncologist. 

The aim of oncology care is to improve survival while preserving or even improving the patients’ QOL by making the correct therapeutic choices. What matters to the patient is (i) if a cure is a realistic option and (ii) how QOL, in all its facets, will evolve upon engaging in therapy. From the viewpoint of the patient, these outcomes matter [16] regardless of the technical details about tumor type, molecular signature of the cancer, or even its stage. Given the drawbacks of the performance score, the QLQ-C30 holds promise as an interesting tool to assess the QOL based on the input of the patient. However, it is now appreciated as a double-edged sword, on the one hand yielding a detailed and balanced image of the patient’s QOL, but on the other hand, hard to implement and interpret because of its multi-dimensionality. Here is where the development of the ‘summarizing’ QSS comes in. With QSS, the analytic challenge because of multiple outcomes generated by the QLQ-C30 and the risk of type I errors due to multiple testing are harnessed [11]. As such, QSS next to GHS might be promising to gauge and monitor the QOL and health of patients being treated for thoracic malignancies. 

Our data give a sobering image of what happens when a patient with a thoracic malignancy is treated with curative or palliative intent. In line with others, we document a baseline difference in health status between both groups, with the former starting systematically better as compared to the latter [17]. The current study gives, in an unprecedented way, an integrated image of what happens to both groups once cancer therapy has started. The curative group shows an exclusively worsening of QOL over one year, based on both GHS and QSS (including most of its dimensions). In a series of resected lung cancer, it was described that surgery substantially reduced QOL across all dimensions except emotional functioning [18]—a finding that parallels our observation in the curative group. In addition, long-lasting deterioration of QOL after therapy with curative intent for patients with lung cancer has been described for subgroups of patients with thoracic malignancies [17,19,20,21,22,23,24,25]. It was recently described that the prevalence of pain in patients with different cancer types, including lung cancer, is disturbing [26,27]. Our data are in line with this finding, and we learn that pain remains a major issue for the patient treated with curative intent. Ongoing attention and improved educational programs on cancer pain management are thus needed, in addition to the fact that thoracic oncologists should proactively probe for this symptom. For patients agreeing to therapy with curative intent, our data might be revealing. They suggest a role for rehab programs to counter the effects on physical health. They also suggest a role for peer associations of patients treated with curative intent to cope with the negative consequences of social isolation. Regardless of the stage of the disease, it was shown that interventions promoting positive, constructive, and problem-oriented strategies, especially in those with a lower quality of life at baseline [28] or at risk for malnutrition [29], are helpful. One should, however, keep in mind our findings are based on a grouping of several therapies encompassing surgery w/wo adjuvant therapies, radiation, and multimodality therapy, all differently affecting QOL. Our data do not have the power to make a detailed difference between these. Furthermore, extrapolation beyond one year is hazardous, as we collected QOL data until that time point only.

Analysis of the evolution of the QOL in the group being treated with palliative intent gives another image. Here, we found mainly a stabilization and a few improvements in the different QOL dimensions. The quality of health and symptom burden is worse at the start of palliative intent therapy [30], making room for improvement larger. Assessment of QOL in stage IV (lung) cancer patients has become standard in clinical trials over the past years. In line with our data, these assessments often show stabilization or improvement of health quality [31]. Patients in clinical trials are, however, highly selected and might be be less representative of the real world. Our data encompass many combinations of systemic treatment schemes with or without palliative radiation therapy or surgery. Differences in the impact on QOL have been described, for example, between targeted therapy and immunotherapy, both doing better than chemotherapy [31,32,33]. However, the overall message remains that palliative therapy retains QOL in addition to yielding a gain in survival. The role of early palliative care in this observation has been shown before [34]. All patients in our study were followed by a multidisciplinary team that consisted of physicians (including specialists with a certification in palliative care) and advanced-practice nurses. Additional consultations with social workers, a dietitian, or a psychologist were offered as soon as the diagnosis was made—regardless of the stage of the disease or the type of therapy. Although we did not measure these visits, we presume that for patients in the palliative setting, these were more frequent as compared to those in the curative setting. It, therefore, seems reasonable to believe that those contacts also in our study might have contributed to the well-being observed in the palliative care setting.

Cancer type, stage, smoking status, gender, and the WHO status are independent prognostic factors for survival in lung cancer [35]. Although less intuitively, some data indicated a role for the patient-reported health status at baseline in different groups of (lung) cancer patients [36,37,38,39,40,41,42]. Recently, a prognostic role for the QSS has been suggested for several cancer types [43], although lung cancer patients were not involved. We found that in addition to therapeutic strategy, the occurrence of severe non-hematologic toxicity causing unforeseen hospitalization was independently prognostic for survival. The patient reported health status (based on GHS or QSS) was not, nor was performance score, age, or comorbidity burden. Whether the heterogeneity of our study population encompassing both palliative and curative strategies and different thoracic malignancies contributes to this observation remains unknown. 

Estimation of the QOL one year after the cancer diagnosis remains an intriguing challenge, since it might be very informative for all stakeholders involved. Scarce attempts in different cancer types can be found wherein baseline patient-reported outcomes are evaluated to predict QOL one year after diagnosis [44,45]. For surgically treated lung cancer, a recent report evaluated the role of a baseline QOL [21]. The authors found that in addition to pre-surgery QOL status, other factors, including perioperative complications, type of surgery, and age, were significant predictors of the QOL. Our series embraces both palliative and curative patients, but the results confirm that the baseline QOL measurements are valuable in the estimation of health one year after therapy starts. The impact of these predictors is considerable since a 1–point change (range 0–100) in baseline QSS implicates nearly 0.5 and 0.3-point change for QSS and GHS after one year, respectively. Remarkably, the WHO performance score, age, and even toxicity were not contributive. Although performance score correlates with GHS and QSS, its small range categorical nature probably explains its failure to predict QOL. 

The current analysis is based on a group of consecutive patients starting therapy for mainly non-small cell and small cell lung cancer. We opted to perform the analysis on a group of all thoracic malignancies so we obtain insights applicable to our case mix, but one has to realize the external validity of our findings needs confirmation in larger groups. In addition, we lumped together different therapeutic strategies into only a curative and palliative group. Although this makes sense from the patient’s viewpoint, one should be aware that the obtained results would benefit from a more in-depth analysis—e.g., when comparing the effects of targeted therapy vs. immunotherapy vs. chemotherapy [32,46,47] or for surgery vs. radiation therapy [48]. In a recent review, it was shown that across most studies, QOL remained stable after treatment with stereotactic radiation therapy, but not after surgery [48]. Finally, we here focused on the role of the QSS because of its comprehensive and analytical advantages. However, the inherent drawback is that details available in the QLQ-C30 are no longer recognized. For example, the emergence of pain or severe social deprivation in patients treated with curative intent needs targeting attention to realize improvements in overall health scores. Whether recurrent measurements are of added values remains debatable since we did observe at most time points similar trends for the different QOL dimensions in the studied groups. This also supports the idea that the baseline measurement of QOL is more important than a high number of follow-up measurements, especially in non-clinical trial settings. 

## 5. Conclusions

We found that patients with thoracic malignancies treated with curative intent are characterized by a worsening of their QOL in the first year after the intervention, in contrast to those receiving palliative therapy. QOL one year after diagnosis depends on the baseline health scores as determined by the patient, so assessment of the QHS and QSS warrants further evaluation in patients with thoracic malignancies. 

## Figures and Tables

**Table 1:** Study population and clinical case-mix variables. Data are presented as numbers (proportions), and age is presented as median (range). The significance level refers to the comparison between curative and palliative groups. Not Significant (N.S.).

	All (*n* = 375)	Curative Group (*n* = 203)	Palliative Group (*n* = 172)	Significance
Age, median (range)	68.0 (38.0–96.0)	69.0 (41.0–93.0)	67.5 (38.0–96.0)	*p* = N.S.
Sex, male *n* (%)	245 (65.3)	140 (69.0)	105 (61.0)	*p* = N.S.
BMI, mean (SEM)	25.6 (0.3)	26.4 (0.4)	24.7 (0.3)	*p* = 0.002
WHO, *n* (%)				*p* = 0.02
▪ 0–1	297 (79.2)	170 (83.7)	127 (73.8)	
▪ 2–3	78 (20.8)	33 (16.3)	45 (26.2)	
Smoking, *n* (%)				*p* = N.S.
▪ Active/Ex	324 (86.4)	180 (88.7)	144 (83.7)	
▪ Never	51 (13.6)	23 (11.3)	28 (16.3)	
Cancer type, *n* (%)				*p* < 0.001
▪ NSCLC	298 (79.5)	150 (73.9)	148 (86.0)	
▪ SCLC	35 (9.3)	17 (8.4)	18 (10.5)	
▪ Other/No Pathol Proof	42 (11.2)	36 (17.7)	6 (3.5)	
Comorbidities, *n* (%)				*p* = N.S.
▪ 0–1	138 (36.8)	64 (31.5)	74 (43.0)	
▪ 2–3	147 (39.2)	84 (41.4)	63 (36.6)	
▪ ≥4	90 (24.0)	55 (27.1)	35 (20.3)	
Stage (clinical), *n* (%)				*p* < 0.001
▪ I	97 (25.9)	97 (47.8)	-	
▪ II	25 (6.7)	25 (12.3)	-	
▪ III	88 (23.5)	81 (39.9)	7 (4.1)	
▪ IV	165 (44.0)	-	165 (95.9)	

## Data Availability

The raw data of this study are available in the department of thoracic oncology, OLV Aalst, and can be provided on request.

## Supplementary Materials

The following supporting information can be downloaded at: https://www.mdpi.com/article/10.3390/cancers15153821/s1, Table S1. Evolution of QSS, GHS, Function Scores (upper panel) and Symptom burden (lower panel) over time.

## References

[1] Hendriks L.E., Kerr K.M., Menis J., Mok T.S., Nestle U., Passaro A., Peters S., Planchard D., Smit E.F., Solomon B.J. (2023). Non-oncogene-addicted metastatic non-small-cell lung cancer: ESMO Clinical Practice Guideline for diagnosis, treatment and follow-up. Ann. Oncol..

[2] Remon J., Soria J.C., Peters S. (2021). Early and locally advanced non-small-cell lung cancer: An update of the ESMO Clinical Practice Guidelines focusing on diagnosis, staging, systemic and local therapy. Ann. Oncol..

[3] Garinet S., Wang P., Mansuet-Lupo A., Fournel L., Wislez M., Blons H. (2022). Updated Prognostic Factors in Localized NSCLC. Cancers.

[4] Metzenmacher M., Griesinger F., Hummel H.D., Elender C., Schäfer H., de Wit M., Kaiser U., Kern J., Jänicke M., Spring L. (2023). Prognostic factors in nonsmall cell lung cancer: Insights from the German CRISP registry. Eur. Respir. J..

[5] Goldstraw P., Chansky K., Crowley J., Rami-Porta R., Asamura H., Eberhardt W.E., Nicholson A.G., Groome P., Mitchell A., Bolejack V. (2016). The IASLC Lung Cancer Staging Project: Proposals for Revision of the TNM Stage Groupings in the Forthcoming (Eighth) Edition of the TNM Classification for Lung Cancer. J. Thorac. Oncol..

[6] Prasad K.T., Kaur H., Muthu V., Aggarwal A.N., Behera D., Singh N. (2018). Interconversion of two commonly used performance tools: An analysis of 5844 paired assessments in 1501 lung cancer patients. World J. Clin. Oncol..

[7] Maltoni M., Amadori D. (2002). Prognosis in advanced cancer. Hematol. Oncol. Clin. N. Am..

[8] Ando M., Ando Y., Hasegawa Y., Shimokata K., Minami H., Wakai K., Ohno Y., Sakai S. (2001). Prognostic value of performance status assessed by patients themselves, nurses, and oncologists in advanced non-small cell lung cancer. Br. J. Cancer.

[9] Kelly C.M., Shahrokni A. (2016). Moving beyond Karnofsky and ECOG Performance Status Assessments with New Technologies. J. Oncol..

[10] Blagden S.P., Charman S.C., Sharples L.D., Magee L.R., Gilligan D. (2003). Performance status score: Do patients and their oncologists agree?. Br. J. Cancer.

[11] Giesinger J.M., Kieffer J.M., Fayers P.M., Groenvold M., Petersen M.A., Scott N.W., Sprangers M.A.G., Velikova G., Aaronson N.K. (2016). Replication and validation of higher order models demonstrated that a summary score for the EORTC QLQ-C30 is robust. J. Clin. Epidemiol..

[12] Sangha O., Stucki G., Liang M.H., Fossel A.H., Katz J.N. (2003). The Self-Administered Comorbidity Questionnaire: A new method to assess comorbidity for clinical and health services research. Arthritis Rheum..

[13] Mak K.S., van Bommel A.C., Stowell C., Abrahm J.L., Baker M., Baldotto C.S., Baldwin D.R., Borthwick D., Carbone D.P., Chen A.B. (2016). Defining a standard set of patient-centred outcomes for lung cancer. Eur. Respir. J..

[14] Maringwa J., Quinten C., King M., Ringash J., Osoba D., Coens C., Martinelli F., Reeve B.B., Gotay C., Greimel E. (2011). Minimal clinically meaningful differences for the EORTC QLQ-C30 and EORTC QLQ-BN20 scales in brain cancer patients. Ann. Oncol..

[15] Di Maio M. (2017). Quality of life: An important element of treatment value. Lancet Oncol..

[16] Porter M.E. (2009). A strategy for health care reform--toward a value-based system. N. Engl. J. Med..

[17] Lehto R.H. (2016). Symptom burden in lung cancer: Management updates. Lung Cancer Manag..

[18] Kenny P.M., King M.T., Viney R.C., Boyer M.J., Pollicino C.A., McLean J.M., Pollicino C.A., McLean J.M., Fulham M.J., McCaughan B.C. (2008). Quality of life and survival in the 2 years after surgery for non small-cell lung cancer. J. Clin. Oncol..

[19] Hirpara D.H., Gupta V., Davis L.E., Zhao H., Hallet J., Mahar A.L., Sutradhar R., Doherty M., Louie A.V., Kidane B. (2020). Severe symptoms persist for Up to one year after diagnosis of stage I-III lung cancer: An analysis of province-wide patient reported outcomes. Lung Cancer.

[20] Li W.W., Lee T.W., Yim A.P. (2004). Quality of life after lung cancer resection. Thorac. Surg. Clin..

[21] Marzorati C., Mazzocco K., Monzani D., Pavan F., Casiraghi M., Spaggiari L., Monturano M. (2020). One-Year Quality of Life Trends in Early-Stage Lung Cancer Patients After Lobectomy. Front. Psychol..

[22] Pompili C. (2015). Quality of life after lung resection for lung cancer. J. Thorac. Dis..

[23] Yucel B., Akkaş E.A., Okur Y., Eren A.A., Eren M.F., Karapinar H., Babacan N.A., Kılıçkap S. (2014). The impact of radiotherapy on quality of life for cancer patients: A longitudinal study. Support Care Cancer.

[24] Nguyen P.A.H., Vercauter P., Verbeke L., Beelen R., Dooms C., Tournoy K.G. (2019). Health Outcomes for Definite Concurrent Chemoradiation in Locally Advanced Non-Small Cell Lung Cancer: A Prospective Study. Respiration.

[25] Yang P., Cheville A.L., Wampfler J.A., Garces Y.I., Jatoi A., Clark M.M., Cassivi S.D., Midthun D.E., Marks R.S., Aubry M.-C. (2012). Quality of life and symptom burden among long-term lung cancer survivors. J. Thorac. Oncol..

[26] Haenen V., Evenepoel M., De Baerdemaecker T., Meeus M., Devoogdt N., Morlion B., Dams L., Van Dijck S., Van der Gucht E., De Vrieze T. (2022). Pain prevalence and characteristics in survivors of solid cancers: A systematic review and meta-analysis. Support Care Cancer.

[27] Snijders R.A.H., Brom L., Theunissen M., van den Beuken-van Everdingen M.H.J. (2023). Update on Prevalence of Pain in Patients with Cancer 2022: A Systematic Literature Review and Meta-Analysis. Cancers.

[28] Chabowski M., Jankowska-Polańska B., Lomper K., Janczak D. (2018). The effect of coping strategy on quality of life in patients with NSCLC. Cancer Manag. Res..

[29] Polański J., Dudek K., Mazur G., Chabowski M. (2023). Effect of nutritional status on psychological functioning and coping in patients with lung cancer. Nutrition.

[30] Tjong M.C., Doherty M., Tan H., Chan W.C., Zhao H., Hallet J., Darling G., Kidane B., Wright F.C., Mahar A. (2021). Province-Wide Analysis of Patient-Reported Outcomes for Stage IV Non-Small Cell Lung Cancer. Oncologist.

[31] Van Der Weijst L., Lievens Y., Schrauwen W., Surmont V. (2019). Health-Related Quality of Life in Advanced Non-small Cell Lung Cancer: A Methodological Appraisal Based on a Systematic Literature Review. Front. Oncol..

[32] Brahmer J.R., Rodríguez-Abreu D., Robinson A.G., Hui R., Csőszi T., Fülöp A., Gottfried A., Peled N., Tafreshi A., Cuffe S. (2017). Health-related quality-of-life results for pembrolizumab versus chemotherapy in advanced, PD-L1-positive NSCLC (KEYNOTE-024): A multicentre, international, randomised, open-label phase 3 trial. Lancet Oncol..

[33] Thongprasert S., Duffield E., Saijo N., Wu Y.L., Yang J.C., Chu D.T., Liao M., Chen Y.M., Kuo H.P., Negoro S. (2011). Health-related quality-of-life in a randomized phase III first-line study of gefitinib versus carboplatin/paclitaxel in clinically selected patients from Asia with advanced NSCLC (IPASS). J. Thorac. Oncol..

[34] Temel J.S., Greer J.A., Muzikansky A., Gallagher E.R., Admane S., Jackson V.A., Dahlin C.M., Blinderman C.D., Jacobsen J.P., William F. (2010). Early palliative care for patients with metastatic non-small-cell lung cancer. N. Engl. J. Med..

[35] Kawaguchi T., Takada M., Kubo A., Matsumura A., Fukai S., Tamura A., Saito R., Maruyama Y., Kawahara M., Ou S.H.L. (2010). Performance status and smoking status are independent favorable prognostic factors for survival in non-small cell lung cancer: A comprehensive analysis of 26,957 patients with NSCLC. J. Thorac. Oncol..

[36] Li T.C., Li C.I., Tseng C.H., Lin K.S., Yang S.Y., Chen C.Y., Hsia T.C., Lee Y.D., Lin C.C. (2012). Quality of life predicts survival in patients with non-small cell lung cancer. BMC Public Health.

[37] Yun Y.H., Kim Y.A., Sim J.A., Shin A.S., Chang Y.J., Lee J., Kim M.S., Shim Y.M., Zo J. (2016). Prognostic value of quality of life score in disease-free survivors of surgically-treated lung cancer. BMC Cancer.

[38] Quinten C., Coens C., Mauer M., Comte S., Sprangers M.A., Cleeland C., Osoba D., Bjordal K., Bottomley A. (2009). Baseline quality of life as a prognostic indicator of survival: A meta-analysis of individual patient data from EORTC clinical trials. Lancet Oncol..

[39] Jacot W., Colinet B., Bertrand D., Lacombe S., Bozonnat M.C., Daurès J.P., Pujol J.L. (2008). Quality of life and comorbidity score as prognostic determinants in non-small-cell lung cancer patients. Ann. Oncol..

[40] Fiteni F., Vernerey D., Bonnetain F., Vaylet F., Sennélart H., Trédaniel J., Moro-Sibilot D., Herman D., Laizé H., Masson P. (2016). Prognostic value of health-related quality of life for overall survival in elderly non-small-cell lung cancer patients. Eur. J. Cancer.

[41] Efficace F., Bottomley A., Smit E.F., Lianes P., Legrand C., Debruyne C., Schramel F., Smit H.J., Gaafar R., Biesma B. (2006). Is a patient’s self-reported health-related quality of life a prognostic factor for survival in non-small-cell lung cancer patients? A multivariate analysis of prognostic factors of EORTC study 08975. Ann. Oncol..

[42] Hong Y.J., Han S., Lim J.U., Kang H.S., Kim S.K., Kim J.W., Lee S.H., Kim S.J., Yeo C.D. (2023). Association Between Quality of Life Questionnaire at Diagnosis and Survival in Patients With Lung Cancer. Clin. Lung Cancer.

[43] Husson O., de Rooij B.H., Kieffer J., Oerlemans S., Mols F., Aaronson N.K., van der Graaf W.T.A., van de Poll-Franse L.V. (2020). The EORTC QLQ-C30 Summary Score as Prognostic Factor for Survival of Patients with Cancer in the “Real-World”: Results from the Population-Based PROFILES Registry. Oncologist.

[44] Lazarewicz M.A., Wlodarczyk D., Johansen Reidunsdatter R. (2023). Decision Tree Analyses for Prediction of QoL over a One-Year Period in Breast Cancer Patients: An Added Value of Patient-Reported Outcomes. Cancers.

[45] Lehto U.S., Ojanen M., Kellokumpu-Lehtinen P. (2005). Predictors of quality of life in newly diagnosed melanoma and breast cancer patients. Ann. Oncol..

[46] Ramirez R.A., Lu J., Thomas K.E.H. (2018). Quality of life for non-small cell lung cancer patients in the age of immunotherapy. Transl. Lung Cancer Res..

[47] Liu W., Zhang Q., Zhang T., Li L., Xu C. (2022). Quality of life in patients with non-small cell lung cancer treated with PD-1/PD-L1 inhibitors: A systematic review and meta-analysis. World J. Surg. Oncol..

[48] Iovoli A.J., Yu B., Ma S.J., Farrugia M.K., Dexter E.U., Yendamuri S., Bouchard E.G., Singh A.K. (2022). Quality of Life after Stereotactic Body Radiation Therapy or Surgery for Early-Stage NSCLC: A Systematic Review. JTO Clin. Res. Rep..

