# Supplementary material for: Health Outcomes with Curative and Palliative Therapies in Real World: Role of the Quality of Life Summary Score in Thoracic Oncology Patients
Source: Cancers (Basel). 2023 Jul 27;15(15):3821. doi: 10.3390/cancers15153821 (PMC10417517; doi:10.3390/cancers15153821)
Supplement: Supplementary file 1 [file cancers-15-03821-s001.zip › cancers-2490888-supplementary.pdf]

Supplementary Material:

Table S1: Evolution of QSS, GHS, Function Scores (upper panel) and Symptom burden (lower panel) over time

Δ mean (SEM)	6 weeks (n = 202)	3 months (n = 252)	6 months (n = 231)	9 months (n = 155)	12 months (n = 201)
Curative					
QSS	-6.9 (1.2)*	-5.4 (1.1)*	-5.0 (1.1)*	-5.0 (1.3)*	-4.5 (1.1)*
GHS	-5.3 (1.9)*	-6.1 (1.8)*	-5.8 (1.8)*	-4.1 (2.1)	-5.1 (1.7)*
Physical functioning	-7.3 (1.8)*	-8.2 (1.5)*	-8.8 (1.5)*	-7.5 (1.9)*	-7.3 (1,7)*
Role functioning	-15.5 (2.8)*	-9.7 (2.6)*	-13.0 (2.8)*	- 7.7 (3.1)	-9.7 (2.8)*
Emotional functioning	+1.7 (2.1)	-1.6 (2.0)	-1.2 (1.7)	-0.4 (2.3)	-1.9 (2.0)
Cognitive functioning	-5.3 (1.7)*	-5.1 (1.6)*	-7.0 (1.6)*	-6.8 (2.1)*	-6.2 (1.7)*
Social functioning	-8.7 (2.4)*	-7.7 (2.1)*	-10.2 (2.0)*	-11.3 (2.7)*	-9.6 (2.2)*
Palliative					
QSS	+2.5 (2.1)	+5.1 (2.2)	+3.6 (2.3)	+4.1 (3.7)	+4.6 (2.8)
GHS	+6.3 (3.7)	+12.8 (3.1)*	+7.2 (3.6)	+8.5 (5.3)	+7.2 (3.9)
Physical functioning	-3.2 (3.0)	+0.3 (3.1)	-0.9 (3.2)	-6.5 (5.6)	-3.3 (3.7)
Role functioning	-1.6 (4.2)	+5.5 (4.6)	-1.5 (4.6)	+0.8 (7.6)	+1.3 (5.1)
Emotional functioning	+8.8 (2.6)*	+11.6 (2.6)*	+12.7 (3.3)*	+9.8 (4.0)	+10.8 (3.4)*
Cognitive functioning	+4.1 (3.2)	-0.4 (2.8)	+1.0 (2.9)	+0.4 (4.8)	+4.0 (3.6)
Social functioning	-6.5 (3.9)	+3.5 (4.2)	+1.2 (3.9)	+1.7 (5.9)	+4.7 (4.7)

Δ mean (SEM)	6 weeks (n = 202)	3 months (n = 252)	6 months (n = 231)	9 months (n = 155)	12 months (n = 201)
Curative					
Fatigue	+14.7 (2.5)*	+10.8 (2.1)*	+9.8 (2.0)*	+7.7 (2.4)*	+7.3 (2.1)*
Pain	+10.2 (2.3) *	+8.6 (2.2) *	+10.0 (2.3)*	+11.4 (2.5)*	+11.9 (2.6)*
Dyspnea	+ 5.4 (2.8)	+4.3 (2.4)	+5.9 (2.6)	+5.8 (2.9)	+8.6 (2.6)*
Cough	+5.7 (2.3)	+5.1 (2.5)	+3.1 (2.3)	+2.0 (2.6)	+ 0.9 (2.1)
Sleep disorder	+1.3 (3.0)	+2.4 (2.7)	-0.2 (2.8)	+2.0 (3.3)	-4.9 (2.9)
Nausea	+3.2 (1.4)	+2.6 (1.2)	+0.4 (1.2)	+0.4 (1.6)	+2.6 (1.4)
Appetite loss	+11.6 (2.8)*	+3.7 (2.4)	-2.9 (2.3)	+1.4 (2.6)	+0.2 (2.2)
Dysphagia	+2.6 (1.8)	+0.8 (2.0)	+0.6 (1.7)	+0.0 (1.8)	+0.9 (1.7)
Palliative					
Fatigue	-3.3 (3.7)	-6.6 (3.7)	-5.9 (3.9)	-6.7 (6.5)	-5.3 (5.0)
Pain	-6.4 (4.3)	-15.0 (4.7)*	-10.8 (4.6)	-11.7 (6.5)	-9.7 (5.9)
Dyspnea	-4.1 (4.3)	-6.1 (3.8)	-6.4 (4.5)	-4.2 (6.6)	-4.7 (5.1)
Cough	+0.9 (3.9)	+0.8 (3.7)	-2.0 (3.4)	-2.5 (4.0)	-6.7 (3.4)
Sleep disorder	-13.2 (3.9)*	-16.3 (4.3)*	-16.2 (5.1)*	-18.3 (5.7)*	-8.7 (5.3)
Nausea	+1.6 (2.0)	-2.4 (2.3)	+0.5 (2.9)	-0.8 (2.7)	-3.0 (3.6)
Appetite loss	-7.8 (4.0)	-6.5 (4.2)	+0.0 (5.1)	-7.5 (6.8)	-10.0 (5.4)
Dysphagia	+1.4 (2.9)	+5.7 (3.8)	+5.9 (4.3)	+4.2 (5.2)	+ 6.0 (4.6)

Data are means (±SEM) and represent the difference from baseline values, significant changes are indicated with an asterix. A red box indicates a significant worsening of QOL. A green box indicates a significant improvement of QOL. Statistical analysis was done using paired T-tests with Bonferroni correction. Figures are based on a case by case exclusion in case of missing values. In the first year of the project, only patients with curative intent were included in the QOL measurements at baseline, 3, 6 and 12 months. Later, all patients were included and 2 additional QOL assessments (6 weeks and 9 months) were also included. This explains why there were fewer measurements at 6 weeks and 9 months.
